# The Modified HZ Conjugate Gradient Algorithm for Large-Scale Nonsmooth Optimization

**DOI:** 10.1371/journal.pone.0164289

**Published:** 2016-10-25

**Authors:** Gonglin Yuan, Zhou Sheng, Wenjie Liu

**Affiliations:** 1 Guangxi Colleges and Universities Key Laboratory of Mathematics and Its Applications, College of Mathematics and Information Science, Guangxi University, Nanning, Guangxi 530004, China; 2 College of Mathematics and Information Science, Guangzhou University, Guangzhou, Guangdong 510006, China; 3 School of Computer and Software, Nanjing University of Information Science & Technology, Nanjing 210044, China; 4 Jiangsu Engineering Center of Network Monitoring, Nanjing University of Information Science & Technology, Nanjing 210044, China; University of Ulm, GERMANY

## Abstract

In this paper, the Hager and Zhang (HZ) conjugate gradient (CG) method and the modified HZ (MHZ) CG method are presented for large-scale nonsmooth convex minimization. Under some mild conditions, convergent results of the proposed methods are established. Numerical results show that the presented methods can be better efficiency for large-scale nonsmooth problems, and several problems are tested (with the maximum dimensions to 100,000 variables).

## Introduction

Consider the following optimization problem:

$$\begin{array}{c} \min\;\;f(x),\;\;x\in \Phi . \end{array}$$ (1)

If the constrained set satisfies Φ = ℜ^*n*^, then Eq (1) is called unconstrained optimization; if Φ = {*x* ∣ *l* ≤ *x* ≤ *u*, *x* ∈ ℜ^*n*^}, where *n* is the number of variables and the vectors *l* and *u* represent the lower and upper bounds on the variables, then Eq (1) is called box-constrained optimization; and if Φ = {*x* ∣ *h*_*i*_(*x*) = 0, *g*_*j*_(*x*) ≤ 0, *x* ∈ ℜ^*n*^, *i* = 1, ⋯, *r*, *j* = 1, ⋯, *k*}, where *r* and *k* are positive integers, then Eq (1) is called normal constrained optimization. If the objective function *f*: ℜ^*n*^ → ℜ^*m*^ is continuously differentiable, then Eq (1) is called smooth optimization; if *f*: ℜ^*n*^ → ℜ^*m*^ is a nondifferentiable function, then Eq (1) is called nonsmooth optimization. For a given objective function *f* and constrained set Φ, Eq (1) will be called the corresponding optimization problem.

Optimization problems are encountered in many fields, including engineering, management, finance, medicine, and biology, and similarly, optimization models can be used in many fields (see [1–13]). At present, there are many efficient methods available for solving optimization problems [14–24]. However, many challenging optimization problems exist, for example, large-scale problems and nonsmooth problems. The workload increases greatly as the dimension of the problem increases, causing the required CPU time to become very long. For certain large-scale problems, a computer may fail to solve them. To address this issue, more efficient algorithms should be designed. It is well known that spectral gradient approaches, conjugate gradient (CG) techniques and limited-memory quasi-Newton methods can cope with large-scale optimization problems. CG methods in particular have been widely used in many practical large-scale optimization problems because of their simplicity and low memory requirements.

Nonsmooth problems are believed to be very difficult to solve, even when they are unconstrained. The direct application of smooth gradient-based methods to nonsmooth problems may lead to a failure in optimality conditions, convergence, or gradient approximation [25]. Haarala et al. (see, e.g., [26, 27]) presented limited-memory bundle methods for large-scale nonsmooth unconstrained and constrained minimization and demonstrated their application to test problems of up to one thousand variables in dimension. Karmitsa et al. [28] tested and compared various methods of both types as well as several methods that may be regarded as hybrids of these two approaches and/or others; the dimensions of the tested nonsmooth problems ranged from 20 to 4000, and the most effective method for large- and extra-large-scale problems was found to be that of [27]. Therefore, special tools for solving nonsmooth optimization problems are needed.

This paper is organized as follows. In the next section, the Hager and Zhang (HZ) CG method is presented, and a modified HZ (MHZ) CG formula is proposed. In Section 3, the application of the HZ and MHZ methods to large-scale nonsmooth problems is discussed, global convergence is established, and numerical experiments on nonsmooth problems are reported. In the last section, conclusions are presented. Throughout the paper, ‖⋅‖ denotes the Euclidean norm.

## The HZ CG formula [29, 30] and a modification thereof

For convenience, we rewrite Eq (1) as the following special case:

$$\begin{array}{c} \min\;\;f\left(x\right),\;\;x\in {ℜ}^{n}, \end{array}$$ (2)

where *f*: ℜ^*n*^ → ℜ is continuously differentiable; Eq (2) is called an unconstrained optimization problem. CG methods are a class of effective line search methods for solving Eq (2), especially when the dimension *n* is large. The iterative formula for CG methods is defined as follows:

$$\begin{array}{c} x_{k+1}=x_{k}+\alpha_{k}d_{k},\;\;k=1,\;2,\cdots , \end{array}$$ (3)

where *x*_*k*_ is the current point in the iteration, *α*_*k*_ > 0 is the step length, and *d*_*k*_ is the search direction; the latter is determined as

$$\begin{array}{c} d_{k+1}=\{\begin{array}{cc} -g_{k+1}+\beta_{k}d_{k}, & \text{if}\;\;\;\;k\geq 1\\ -g_{k+1}, & \text{if}\;\;\;\;k=0, \end{array} \end{array}$$ (4)

where *g*_*k*+1_ = ∇*f*(*x*_*k*+1_) is the gradient of *f*(*x*) at point *x*_*k*+1_ and *β*_*k*_ ∈ ℜ is a scalar. The parameter *β*_*k*_ is chosen such that when the method is applied to minimize a strongly convex quadratic function, the directions *d*_*k*_ and *d*_*k*−1_ are conjugate with respective to the Hessian of the quadratic function. Let $\beta_{k}=\beta_{k}^{N},$ where from [29]

$$\begin{array}{c} \beta_{k}^{N}={\left(y_{k}-2\frac{{∥y_{k}∥}^{2}}{d_{k}^{T}y_{k}}d_{k}\right)}^{T}\frac{g_{k+1}}{d_{k}^{T}y_{k}} \end{array}$$ (5)

with *y*_*k*_ = *g*_*k*+1_ − *g*_*k*_; we call this formula for $\beta_{k}^{N}$ [29] the HZ formula. If $d_{k}^{T}y_{k}\neq 0$, then Eq (4) with $\beta_{k}=\beta_{k}^{N}$ satisfies

$$\begin{array}{c} d_{k+1}^{T}g_{k+1}\leq -\frac{7}{8}{∥g_{k+1}∥}^{2}. \end{array}$$ (6)

This method exhibits global convergence for the strongly convex function *f*. To obtain a similar result for a general nonlinear function, Hager and Zhang [30] proposed the formula

$$\begin{array}{c} {\bar{\beta }}_{k}^{N}=\max\left\{\beta_{k}^{N},\eta_{k}\right\},\;\;\eta_{k}=\frac{-1}{∥d_{k}∥\min\{\eta ,∥g_{k}∥\}}, \end{array}$$

where *η* > 0 is a constant; in their experiments, they set *η* = 0.01. This new parameter ${\bar{\beta }}_{k}^{N}$ also has the property expressed in Eq (6).

Based on the formula for $\beta_{k}^{N}$, if we let $T_{k}=d_{k}^{T}y_{k}$, we can obtain the more general formula

$$\begin{array}{c} \beta_{k}^{GN}={\left(y_{k}-2\frac{{∥y_{k}∥}^{2}}{T_{k}}d_{k}\right)}^{T}\frac{g_{k+1}}{T_{k}}. \end{array}$$ (7)

In this paper, we set $T_{k}=\max\{c∥d_{k}∥∥y_{k}∥,d_{k}^{T}y_{k},\frac{2{∥y_{k}∥}^{2}d_{k}^{T}g_{k+1}}{y_{k}^{T}g_{k+1}}\},$ where *c* ∈ (0, 1) is a scalar. It is easy to deduce that $T_{k}=\max\{c∥d_{k}∥∥y_{k}∥,d_{k}^{T}y_{k},\frac{2{∥y_{k}∥}^{2}d_{k}^{T}g_{k+1}}{y_{k}^{T}g_{k+1}}\}\geq c∥d_{k}∥∥y_{k}∥\geq 0$; moreover, if $T_{k}=d_{k}^{T}y_{k}$, then Eq (7) is identical to the HZ formula. The modified formula has the following features:

(i) The new formula can overcome the shortcomings of the CG parameter *β*_*k*_. If $y_{k}^{T}g_{k+1}\geq 0$, it is not difficult to find that

$$\begin{array}{ccc} \beta_{k}^{GN} & = & {\left(y_{k}-2\frac{{∥y_{k}∥}^{2}}{T_{k}}d_{k}\right)}^{T}\frac{g_{k+1}}{T_{k}}\\ & = & \frac{y_{k}^{T}g_{k+1}T_{k}-2{∥y_{k}∥}^{2}d_{k}^{T}g_{k+1}}{T_{k}^{2}}\\ & \geq & \frac{y_{k}^{T}g_{k+1}\frac{2{∥y_{k}∥}^{2}d_{k}^{T}g_{k+1}}{y_{k}^{T}g_{k+1}}-2{∥y_{k}∥}^{2}d_{k}^{T}g_{k+1}}{T_{k}^{2}}=0. \end{array}$$ (8)

(ii) Another property of this formula is that it can ensure that the new direction *d*_*k*+1_ in Eq (4) with $\beta_{k}=\beta_{k}^{GN}$ belongs to a trust region without the need for any line search technique. By Eq (4), for $\beta_{k}=\beta_{k}^{GN}$, we obtain

$$\begin{array}{ccc} ∥d_{k+1}∥ & = & ∥-g_{k+1}+\beta_{k}^{GN}d_{k}∥\\ & \leq & ∥g_{k+1}∥+|\beta_{k}^{GN}|∥d_{k}∥\\ & \leq & ∥g_{k+1}∥+∥\left(y_{k}-2\frac{{∥y_{k}∥}^{2}}{T_{k}}d_{k}\right)∥\frac{∥g_{k+1}∥}{T_{k}}∥d_{k}∥\\ & \leq & ∥g_{k+1}∥+\frac{∥y_{k}∥∥g_{k+1}∥+\frac{2{∥y_{k}∥}^{2}∥d_{k}∥∥g_{k+1}∥}{T_{k}}}{T_{k}}∥d_{k}∥\\ & \leq & ∥g_{k+1}∥+\frac{∥y_{k}∥∥g_{k+1}∥+\frac{2{∥y_{k}∥}^{2}∥d_{k}∥∥g_{k+1}∥}{c∥d_{k}∥∥y_{k}∥}}{c∥d_{k}∥∥y_{k}∥}∥d_{k}∥\\ & = & \left(1+\frac{1}{c}+\frac{2}{c^{2}}\right)∥g_{k+1}∥. \end{array}$$ (9)

By combining this result with Step 1 of Algorithm 2.2, we find that $∥d_{k}∥\leq \left(1+\frac{1}{c}+\frac{2}{c^{2}}\right)∥g_{k}∥$ for all *k*.

(iii) If *T*_*k*_ ≠ 0, then the new direction *d*_*k*+1_ in Eq (4) when $\beta_{k}=\beta_{k}^{GN}$ possesses the following sufficient descent property:

$$\begin{array}{c} d_{k+1}^{T}g_{k+1}\leq -\frac{7}{8}{∥g_{k+1}∥}^{2}, \end{array}$$ (10)

which holds for all *k*. Now let us analyze this result. If *k* = 0, we have *d*_1_ = −*g*_1_ and $d_{1}^{T}g_{1}=-{∥g_{1}∥}^{2},$ satisfying Eq (10). For *k* ≥ 1, because *T*_*k*_ ≠ 0, multiplying Eq (4) by $g_{k+1}^{T}$ yields

$$\begin{array}{ccc} g_{k+1}^{T}d_{k+1} & = & -{∥g_{k+1}∥}^{2}+\beta_{k}^{GN}d_{k}^{T}g_{k+1}\\ & = & -{∥g_{k+1}∥}^{2}+d_{k}^{T}g_{k+1}{\left(y_{k}-2\frac{{∥y_{k}∥}^{2}}{T_{k}}d_{k}\right)}^{T}\frac{g_{k+1}}{T_{k}}\\ & = & \frac{y_{k}^{T}g_{k+1}T_{k}\left(d_{k}^{T}g_{k+1}\right)-{∥g_{k+1}∥}^{2}T_{k}^{2}-2{∥y_{k}∥}^{2}\left(d_{k}^{T}g_{k+1}\right)}{T_{k}^{2}}. \end{array}$$ (11)

Let $u=\frac{1}{2}T_{k}g_{k+1}$ and $v=2\left(d_{k}^{T}g_{k+1}\right)y_{k}$; then, by the inequality $u^{T}v\leq \frac{1}{2}\left(u^{2}+v^{2}\right)$, we have

$$\begin{array}{ccc} g_{k+1}^{T}d_{k+1} & \leq & \frac{\frac{1}{2}{(\frac{1}{4}T_{k}^{2}{∥g_{k+1}∥}^{2}+4\left(d_{k}^{T}g_{k+1}\right)}^{2}{∥y_{k}∥}^{2})-{∥g_{k+1}∥}^{2}T_{k}^{2}-2{∥y_{k}∥}^{2}\left(d_{k}^{T}g_{k+1}\right)}{T_{k}^{2}}\\ & = & -\frac{7}{8}{∥g_{k+1}∥}^{2}. \end{array}$$

## Nonsmooth Convex Problems and Their Results

Consider the unconstrained convex optimization problem

$$\begin{array}{c} \min_{x\in {ℜ}^{n}}f\left(x\right), \end{array}$$ (12)

where *f*: ℜ^*n*^ → ℜ is a possibly nonsmooth convex function. For the special case that *f* is continuously differentiable, this optimization problem has been well studied for several decades. However, nonsmooth optimization problems of the form of Eq (12) also arise in many applications, such as image restoration [31] and optimal control [32]. The Moreau-Yosida regularization of *f* generates

$$\begin{array}{c} F\left(x\right)=\min_{z\in {ℜ}^{n}}\left\{f\left(z\right)+\frac{1}{2\lambda }{∥z-x∥}^{2}\right\}, \end{array}$$ (13)

where *λ* is a positive parameter; then, Eq (12) is equivalent to the following problem:

$$\begin{array}{c} \min_{x\in {ℜ}^{n}}F\left(x\right). \end{array}$$ (14)

Let *p*(*x*) = argmin *θ*(*z*) and define a function

$$\begin{array}{c} \theta \left(z\right)=f\left(z\right)+\frac{1}{2\lambda }{∥z-x∥}^{2}. \end{array}$$

Thus, *p*(*x*) is well defined and unique because the function *θ*(*z*) is strongly convex. Therefore, *F*(*x*) can be expressed as follows:

$$\begin{array}{c} F\left(x\right)=f\left(p\left(x\right)\right)+\frac{1}{2\lambda }{∥p(x)-x∥}^{2}. \end{array}$$

*F*(*x*) possesses many known features (see, e.g., [33–35]). The generalized Jacobian of *F*(*x*) and its property of BD-regularity are demonstrated in [36, 37]. Here, we list some additional findings regarding the function *F*(*x*), as follows:

(i) *x* is an optimal solution to Eq (12) ⇔ ∇*F*(*x*) = 0, i.e., *p*(*x*) = *x*.

(ii)

$$\begin{array}{c} ∥g\left(x\right)-g\left(y\right)∥\leq \frac{1}{\lambda }∥x-y∥,\;\;\forall \;\;x,y\in {ℜ}^{n}, \end{array}$$ (15)

where

$$\begin{array}{c} g\left(x\right)=\nabla F\left(x\right)=\frac{x-p(x)}{\lambda } \end{array}$$ (16)

is the gradient of *F*.

(iii) The set of generalized Jacobian matrices ∂_*B*_*g*(*x*) = {*V* ∈ ℜ^*n*×*n*^: *V* = lim_*x*_*k*_ → *x*_∇*g*(*x*_*k*_), *x*_*k*_ ∈ *D*_*g*_} is nonempty and compact, where $D_{g}=\left\{x\in {ℜ}^{n}:g\;\;\text{is}\;\;\text{differentiable}\;\;\text{at}\;\;x\right\}.$

Furthermore, every *V* ∈ ∂_*B*_*g*(*x*) is a symmetric positive semidefinite matrix for each *x* ∈ ℜ^*n*^ because *g* is a gradient mapping of the convex function *F*.

(iv) There exist two constants, *μ*_1_ > 0 and *μ*_2_ > 0, and a neighborhood *Ω* of *x* that satisfy

$$\begin{array}{c} d^{T}Vd\geq \mu_{1}{∥d∥}^{2},\;∥V^{-1}∥\leq \mu_{2},\;\forall \;\;d\in {ℜ}^{n},\;V\in \partial_{B}g\left(x\right). \end{array}$$

## Two algorithms for nonsmooth problems

Based on the above discussion, we present two algorithms for application to nonsmooth problems of the form Eq (14); afterward, we analyze the solution of Eq (12). In the following, unless otherwise noted, *g*_*k*_ = *g*(*x*_*k*_) is defined as in Eq (16).

**Algorithm 1** Algorithm 4.1.

**Require**:

 An initial point *x*_0_ ∈ ℜ^*n*^, *λ* > 0, *σ*, *η* ∈ (0, 1), *ρ* ∈ (0, 1/2], *ϵ* ∈ [0, 1).

 Specify *g*_0_ by solving the subproblem Eq (13);

 *d*_0_ ← −*g*_0_, *k* ← 0.

 **while** ‖*g*_*k*_‖ > *ϵ* **do**

  Determine the step size *α*_*k*_ = max{*ρ*^*j*^|*j* = 0, 1, 2, ⋯} satisfying the following Armijo line search condition

$$\begin{array}{c} F\left(x_{k}+\alpha_{k}d_{k}\right)\leq F\left(x_{k}\right)+\sigma \alpha_{k}g_{k}^{T}d_{k}; \end{array}$$ (17)

  *x*_*k*+1_ = *x*_*k*_ + *α*_*k*_ *d*_*k*_;

  Compute *g*_*k*+1_ by solving the subproblem Eq (13);

  **if** ‖*g*_*k*+1_‖ ≤ *ϵ* **then**

   break.

  **else**

   Compute *d*_*k*_ as

$$\begin{array}{c} d_{k+1}=\{\begin{array}{cc} -g_{k+1}+{\bar{\beta }}_{k}^{N}d_{k}, & \text{if}\;\;\;\;k\geq 1\\ -g_{k+1}, & \text{if}\;\;\;\;k=0 \end{array} \end{array}$$ (18)

  **end if**

  *x*_*k*_ ← *x*_*k*+1_, *d*_*k*_ ← *d*_*k*+1_, *k* ← *k* + 1.

**end while**

**Algorithm 4.2.** Eq (18) of Algorithm 4.1 is replaced with the following: Compute *d*_*k*_ as

$$\begin{array}{c} d_{k+1}=\{\begin{array}{cc} -g_{k+1}+\beta_{k}^{GN}d_{k}, & \text{if}\;\;\;\;k\geq 1\\ -g_{k+1}, & \text{if}\;\;\;\;k=0, \end{array} \end{array}$$ (19)

and let *k*: = *k* + 1, go back to Step 2.

The following assumptions are needed to ensure the global convergence of Algorithms 4.1 and 4.2.

**Assumption 4.1.** (i) *F* is bounded from below and the sequence {*V*_*k*_} is bounded, namely, there exists a constant *M* > 0 such that

$$\begin{array}{c} ∥V_{k}∥\leq M,\;\;\forall \;\;k. \end{array}$$ (20)

(ii) *g* is BD-regular at *x*, namely, item (iv) in Section 3 above holds.

By Assumption 4.1, it is not difficult to deduce that there exists a constant M_*_ > 1 such that

$$\begin{array}{c} ∥d_{k}∥\leq M_{*}∥g_{k}∥,\;\forall k. \end{array}$$ (21)

**Lemma 1** *The sequence* {*x*_*k*_} *is generated by Algorithm 4.1 (or Algorithm 4.2). Let Assumption 4.1 hold; then, for sufficiently large k, there exists a constant* α_*_ > 0 *that satisfies*

$$\begin{array}{c} \alpha_{k}\geq \alpha_{*}. \end{array}$$ (22)

**Proof.** Suppose that *α*_*k*_ satisfies the Armijo line search condition Eq (17). The proof is complete if *α*_*k*_ = 1 holds. Otherwise, let $\alpha_{k}^{\prime }=\frac{\alpha_{k}}{\rho }$; then, we have

$$\begin{array}{c} F\left(x_{k}+\alpha_{k}^{\prime }d_{k}\right)-F\left(x_{k}\right)>\sigma \alpha_{k}^{\prime }g{\left(x_{k}\right)}^{T}d_{k}. \end{array}$$

By performing a Taylor expansion, we obtain

$$\begin{array}{ccc} \sigma \alpha_{k}^{\prime }g{\left(x_{k}\right)}^{T}d_{k} & < & F\left(x_{k}+\alpha_{k}^{\prime }d_{k}\right)-F\left(x_{k}\right)\\ & = & \alpha_{k}^{\prime }d_{k}^{T}g\left(x_{k}\right)+\frac{1}{2}{\left(\alpha_{k}^{\prime }\right)}^{2}d_{k}^{T}V\left(\xi_{k}\right)d_{k}\\ & \leq & \alpha_{k}^{\prime }d_{k}^{T}g\left(x_{k}\right)+\frac{M}{2}{\left(\alpha_{k}^{\prime }\right)}^{2}{∥d_{k}∥}^{2}, \end{array}$$ (23)

where $\xi_{k}=x_{k}+\theta \alpha_{k}^{\prime }d_{k},\;\;\theta \in \left(0,1\right),$ and the last inequality follows from Eq (20). By combining $d_{k}^{T}g_{k}\leq -\frac{7}{8}{∥g_{k}∥}^{2},$ Eqs (21) and (23), we obtain

$$\begin{array}{c} \alpha_{k}^{\prime }>\left[\frac{-\left(1-\sigma \right)g{\left(x_{k}\right)}^{T}d_{k}}{{∥d_{k}∥}^{2}}\right]\frac{2}{M}=\left[\frac{\left(1-\sigma \right){∥g(x_{k})∥}^{2}}{{∥d_{k}∥}^{2}}\right]\frac{7}{4M}\geq \frac{7(1-\sigma )}{4MM_{*}^{2}}. \end{array}$$ (24)

Thus, we find that

$$\begin{array}{c} \alpha_{k}\geq \frac{7\rho (1-\sigma )}{4MM_{*}^{2}}. \end{array}$$

Let $\alpha_{*}\in \left(0,\frac{7\rho (1-\sigma )}{4MM_{*}^{2}}\right]$, and the proof is complete.

Now, let us prove the global convergence of Algorithm 4.1.

**Theorem 1** *Suppose that the conditions in Lemma 1 hold. Then, we have*

$$\begin{array}{c} \lim_{k\to \infty }∥g\left(x_{k}\right)∥=0; \end{array}$$ (25)

*moreover, any accumulation point of* *x*_*k*_ *is an optimal solution of* Eq (12).

**Proof.** Suppose that Eq (25) is not true. Then, there must exist two constants, *ϵ*_0_ > 0 and k_*_ > 0, that satisfy

$$\begin{array}{c} ∥g\left(x_{k}\right)∥\geq \epsilon_{0},\;\;\forall \;k>k_{*}. \end{array}$$ (26)

By combining $d_{k}^{T}g_{k}\leq -\frac{7}{8}{∥g_{k}∥}^{2}$, Eqs (17), (22) and (26), we obtain

$$\begin{array}{c} F\left(x_{k+1}\right)-F\left(x_{k}\right)\leq \sigma \alpha_{k}g{\left(x_{k}\right)}^{T}d_{k}=-\frac{7\sigma }{8}\alpha_{k}{∥g(x_{k})∥}^{2}\leq -\frac{7\sigma \alpha_{*}\epsilon_{0}^{2}}{8},\;\;\forall \;\;k>k_{*}. \end{array}$$ (27)

Because *F*(*x*) is bounded from below for all *k*, it follows from Eq (27) that

$$\begin{array}{c} \sum_{k=k_{0}}^{\infty }\frac{7\sigma \alpha_{*}\epsilon_{0}^{2}}{8}<\infty . \end{array}$$

This contradicts $\sum_{k=k_{0}}^{\infty }\frac{7\sigma \alpha_{*}\epsilon_{0}^{2}}{8}=\infty .$ Therefore, Eq (25) holds. Let x* be an accumulation point of {*x*_*k*_} and, without loss of generality, suppose that there exists a subsequence {*x*_*k*_}_*K*_ that satisfies

$$\begin{array}{c} \lim_{k\in K,\;k\to \infty }x_{k}=x^{*}. \end{array}$$ (28)

From Eq (28), we find that ‖*g*(x*)‖ = ‖∇*F*(x*)‖ = 0. Then, from property (i) of *F*(*x*) as given in Section 3, x* is an optimal solution of Eq (12). The proof is complete.

In a manner similar to Theorem 4.1 in [38], we can establish the linear convergence rate of Algorithm 4.1 (or Algorithm 4.2). Here, we simply state this property, as follows, but omit the proof.

**Theorem 2** *Let Assumptions 4.1 (i) and (ii) hold, and let* x* *be the unique solution of* Eq (14). *Then, there exist two constants* *b* > 0 *and* *r* ∈ (0, 1) *that satisfy*

$$\begin{array}{c} ∥x_{k}-x^{*}∥\leq br^{k}, \end{array}$$ (29)

*namely, the sequence* {*x*_*k*_} *generated by Algorithm 4.1 (or Algorithm 4.2) linearly converges to* x*.

## Numerical results for nonsmooth problems

In this section, we present several numerical experiments using Algorithms 4.1 and 4.2 and a modified Polak-Ribière-Polyak conjugate gradient algorithm (called MPRP) [18]. It is well known that the CG method is very effective for large-scale smooth problems. We will show that these two algorithms are also applicable to large-scale nonsmooth problems. The nonsmooth academic test problems that are listed, along with their initial points, in Table 1 are described in [27], where “Problem” is the name of the test problem, “*x*_0_” is the initial point, and the corresponding numbers of variables are also given, “*f*_*ops*_” is the optimal value of the test problem. Problems 1-5 are convex functions, and the others are nonconvex functions. The detailed characteristics of these problems can be found in [27]. Because we wished to test the three considered methods for application to large-scale nonsmooth problems, problem dimensions of 5000, 10000, 50000, and 100000 were chosen. In our experiments, we found that problem 2 required a considerably amount of time to solve; therefore, we set its largest dimension to 50000.

**Table 1:** Test problems and their initial points and optimal value.

No.	Problem	*x*_0_	*f*_*ops*_
1	Generalization of MAXQ	(1, 2, ⋯, *n*/2, −(*n*/2 + 1), ⋯, −*n*)	0
2	Generalization of MXHILB	(1, 1, ⋯, 1)	0
3	Chained LQ	(−0.5, −0.5, ⋯, −0.5)	$-\sqrt{2(n-1)}$
4	Chained CB3 I	(2, 2, ⋯, 2)	2(*n* − 1)
5	Chained CB3 II	(2, 2, ⋯, 2)	2(*n* − 1)
6	Number of active faces	(1, 0, ⋯, 1, 0)	0
7	Nonsmooth generalization of Brown function 2	(−1, −1, ⋯, −1)	0
8	Chained Mifflin 2	(−1.5, 2, ⋯, −1.5, 2)	varies
9	Chained Crescent I	(1, 0, ⋯, 1, 0)	0
10	Chained Crescent II	(1, 0, ⋯, 1, 0)	0

Both algorithms were implemented using Fortran PowerStation 4.0 with double-precision arithmetic, and all experiments were run on a PC with a Core 2 Duo E7500 CPU @2.93 GHz with 2.00 GB of memory and the Windows XP operating system. The following parameters were chosen for Algorithms 4.1 and 4.2 and MPRP: *σ* = 0.8, *ρ* = 0.5, *c* = 0.01, *ϵ*_*k*_ = 1*E* − 15, and *η* = 0.01. We stopped the algorithms when the condition ‖*g*(*x*)‖ ≤ 1*E* − 5 or ∣*F*(*x*_*k*+1_) − *F*(*x*_*k*_)∣ ≤ 1*E* − 8 or |*f*(*x*_*k*_) − *f*_*ops*_| ≤ 1*E* − 4 was satisfied. If the number of iterations exceeded ten thousand, the program would also terminate. Because a line search cannot always ensure the descent condition $d_{k}^{T}g_{k}<0$, an uphill search direction may arise in real numerical experiments, which may cause the line search rule to fail. To avoid this condition, the step size *α*_*k*_ was accepted only if the search number in the line search was greater than five.

In the experiments, the subproblem Eq (13) was solved using the PRP CG method (called the sub-algorithm), and its numbers of iterations and function evaluations were added to those of Algorithm 4.1, Algorithm 4.2 or MPRP (called the main algorithm). In the sub-algorithm, if ‖∂*f*(*x*_*k*_)‖ ≤ 1*E* − 4 or *f*(*x*_*k*+1_) − *f*(*x*_*k*_) + ‖∂*f*(*x*_*k*+1_)‖^2^ − ‖∂*f*(*x*_*k*_)‖^2^ ≤ 1*E* − 3 (see [39]) holds, where ∂*f*(*x*_*k*_) is the subgradient of *f*(*x*) at point *x*_*k*_, then the algorithm terminates. The sub-algorithm will also terminate when the iteration number exceeds ten. For the line search, the Armijo line search technique was used and the step length was accepted if the search number was greater than five. The columns in Tables 2, 3 and 4 have the following meanings:

Dim: the dimensions of problem.
NI: the total number of iterations.
NF: the number of function evaluations.
‖*g*(*x*)‖: the norm of *g*(*x*) at the final iteration.
Time: the CPU time in seconds.
*f*_*final*_: the value of *f*(*x*) at the final iteration.

**Table 2:** Test results for Algorithm 4.1.

No.	Dim	NI/NF	‖*g*(*x*)‖	Time	*f*_*final*_
1	5000	122 / 2304	4.963444E-05	3.109375E-01	2.977173E-08
1	50000	142 / 2720	5.295883E-04	3.451688E+00	3.177435E-08
1	100000	148 / 2844	8.533346E-04	8.389563E+00	2.559965E-08
2	5000	48 / 902	0	5.279200E+02	0
2	10000	52 / 978	0	2.290124E+03	0
2	50000	63 / 1194	3.689089E-10	1.577677E+04	9.789607E-07
3	3000	11 / 55	6.505226E-16	7.031086E-04	-4.241179E+03
4	3000	5 / 50	0	2.943751E-02	5.998031E+03
5	3000	14 / 65	0	4.543751E-02	5.998000E+03
6	5000	64 / 1240	7.081679E-16	5.470469E-01	1.881710E-06
6	10000	69 / 1345	7.428122E-16	1.187422E+00	2.725454E-06
6	50000	82 / 1618	6.935890E-16	7.172547E+00	5.888909E-06
6	100000	87 / 1723	7.275201E-16	1.548309E+01	8.529442E-06
7	5000	12 / 59	2.710511E-16	2.024688E-01	2.327843E-06
7	10000	13 / 61	5.421024E-16	4.373125E-01	4.656153E-06
7	50000	23 / 82	6.776293E-16	2.733234E+00	1.164132E-05
7	100000	41 / 119	3.388159E-16	8.077172E+00	1.164146E-05
8	3000	12 / 58	6.505227E-16	1.404690E-02	-2.120705E+03
9	5000	16 / 124	5.750809E-16	1.084375E-01	7.202892E-07
9	10000	16 / 125	2.875404E-16	3.114688E-01	7.198567E-07
9	50000	26 / 146	3.594263E-16	3.202235E+00	1.798780E-06
9	100000	27 / 148	7.188527E-16	9.281172E+00	3.597344E-06
10	5000	13 / 61	8.470350E-16	3.274688E-01	4.365231E-06
10	10000	14 / 64	4.235176E-16	5.623125E-01	4.365406E-06
10	50000	42 / 121	5.293999E-16	3.483234E+00	1.091389E-05
10	100000	51 / 140	2.647004E-16	9.937172E+00	1.091395E-05

**Table 3:** Test results for Algorithm 4.2.

No.	Dim	NI/NF	‖*g*(*x*)‖	Time	*f*_*final*_
1	5000	186 / 3878	4.403006E-06	4.539688E-01	2.641011E-09
1	50000	242 / 5051	8.447711E-05	6.235391E+00	5.068475E-09
1	100000	259 / 5377	3.400454E-04	1.389234E+01	1.020121E-08
2	5000	98 / 2010	5.769694E-06	1.189811E+03	3.089375E-04
2	10000	107 / 2199	3.611318E-06	5.205405E+03	1.859985E-04
2	50000	129 / 2679	3.710004E-06	1.349417E+04	9.817318E-05
3	3000	11 / 55	6.505226E-16	1.590626E-02	-4.241179E+03
4	3000	7 / 89	0	2.552654E-02	5.998031E+03
5	3000	16 / 104	0	7.656254E-02	5.998000E+03
6	5000	64 / 1240	7.081679E-16	5.306719E-01	1.881710E-06
6	10000	69 / 1345	7.428122E-16	1.157047E+00	2.725454E-06
6	50000	82 / 1618	6.935890E-16	7.391297E+00	5.888909E-06
6	100000	87 / 1723	7.275201E-16	1.539180E+01	8.529442E-06
7	5000	12 / 42	4.656623E-16	2.339532E-01	2.327843E-06
7	10000	13 / 44	9.313248E-16	4.377500E-01	4.656153E-06
7	50000	23 / 66	2.910396E-16	2.734688E+00	1.164132E-05
7	100000	41 / 102	5.820813E-16	7.891281E+00	1.164146E-05
8	3000	12 / 58	6.505227E-16	3.190626E-02	-2.120705E+03
9	5000	16 / 107	9.879814E-16	2.969532E-01	7.202892E-07
9	10000	16 / 108	4.939907E-16	5.157501E-01	7.198567E-07
9	50000	26 / 129	6.174896E-16	3.202688E+00	1.798780E-06
9	100000	27 / 132	3.087449E-16	9.000281E+00	3.597344E-06
10	5000	13 / 45	3.637988E-16	3.119532E-01	4.365231E-06
10	10000	14 / 47	7.275977E-16	5.627500E-01	4.365406E-06
10	50000	42 / 104	9.095022E-16	3.468688E+00	1.091389E-05
10	100000	51 / 123	4.547519E-16	9.625281E+00	1.091395E-05

**Table 4:** Test results for MPRP.

No.	Dim	NI/NF	‖*g*(*x*)‖	Time	*f*_*final*_
1	5000	250 / 5197	1.146977E-04	6.209688E-01	6.879798E-08
1	50000	286 / 5991	1.099941E-03	8.980437E+00	6.599447E-08
1	100000	297 / 6222	2.127262E-03	2.067906E+01	6.381689E-08
2	5000	98 / 2025	5.373446E-15	1.105497E+03	9.428024E-09
2	10000	107 / 2214	3.363302E-15	4.828827E+03	5.676222E-09
2	50000	129 / 2693	1.382084E-14	1.683294E+04	5.992015E-09
3	3000	11 / 55	6.505226E-16	3.221878E-02	2.793039E-06
4	3000	7 / 89	0	4.721878E-02	7.714992E+03
5	3000	16 / 104	0	7.821879E-02	7.714994E+03
6	5000	64 / 1240	7.081679E-16	6.085625E-01	1.881710E-06
6	10000	69 / 1345	7.428122E-16	1.265188E+00	2.725454E-06
6	50000	82 / 1618	6.935890E-16	7.234313E+00	5.888909E-06
6	100000	87 / 1723	7.275201E-16	1.573519E+01	8.529442E-06
7	5000	12 / 59	2.710511E-16	1.105805E+03	2.327843E-06
7	10000	13 / 61	5.421024E-16	4.829446E+03	4.656153E-06
7	50000	23 / 82	6.776293E-16	1.037413E+01	1.164132E-05
7	100000	41 / 119	3.388159E-16	1.103525E+01	1.164146E-05
8	3000	12 / 58	6.505227E-16	3.259378E-02	-2.120705E+03
9	5000	16 / 124	5.750809E-16	1.105867E+03	7.202892E-07
9	10000	16 / 125	2.875404E-16	4.829565E+03	7.198567E-07
9	50000	26 / 146	3.594263E-16	1.115213E+01	1.798780E-06
9	100000	27 / 148	7.188527E-16	1.286225E+01	3.597344E-06
10	5000	13 / 61	8.470350E-16	1.105892E+03	4.365231E-06
10	10000	14 / 64	4.235176E-16	4.829612E+03	4.365406E-06
10	50000	42 / 121	5.293999E-16	1.158712E+01	1.091389E-05
10	100000	51 / 140	2.647004E-16	1.381325E+01	1.091395E-05

**Fig 1:**
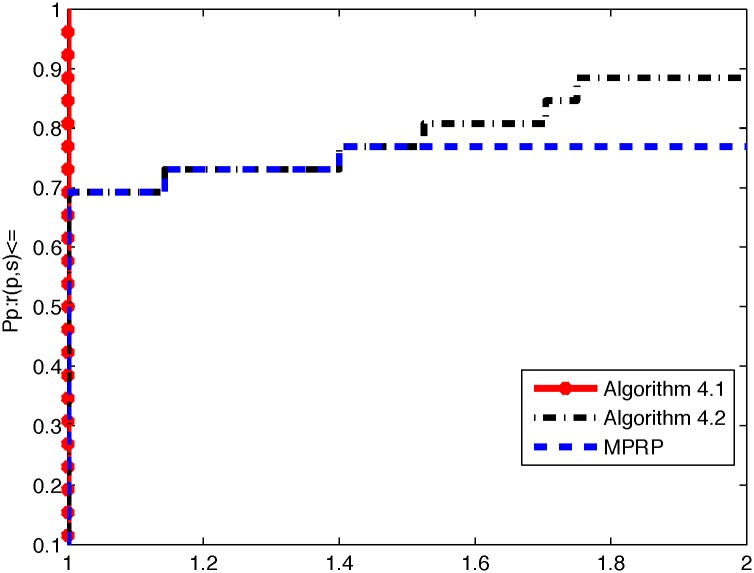
Performance profiles of these methods (NI).

**Fig 2:**
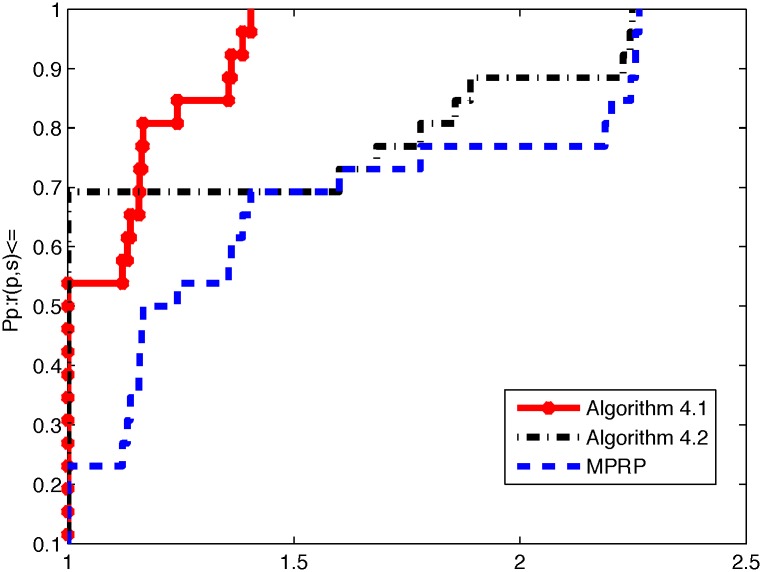
Performance profiles of these methods (NF).

**Fig 3:**
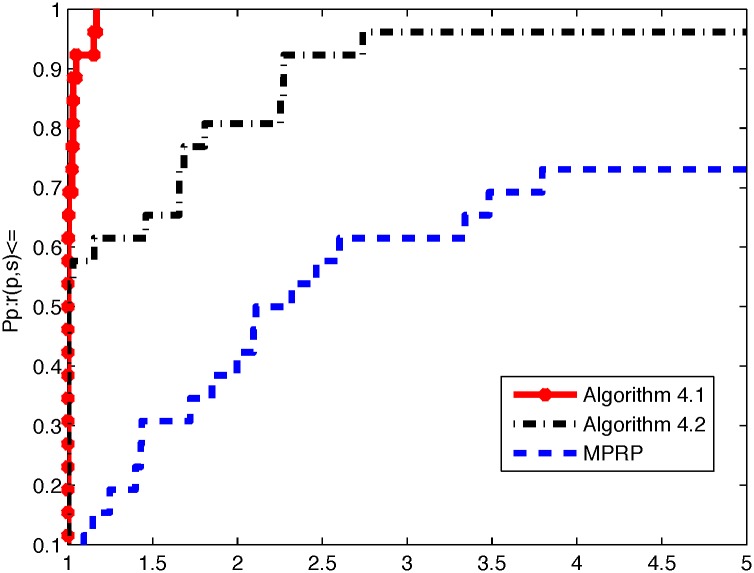
Performance profiles of these methods (Time).

From the above three tables, it is not difficult to see that Algorithm 4.1 is superior to Algorithm 4.2 and MPRP in terms of NI, NF, and ‖*g*(*x*)‖. Algorithm 4.1 yields smaller values of NI and NF when the program terminates; moreover, the value of ‖*g*(*x*)‖ is smaller than that for Algorithm 4.2 in most cases. However, we also note that our algorithms can efficient solve the 3000 dimensional(with maximum dimensional) case for problems 3, 4, 5 and 8, if we increase the dimensional, these algorithms fail to converge to good minima and become stuck at local. To directly illustrate the performances of these two methods, we used the tool developed by Dolan and Moré [40] to analyze their efficiencies in terms of the number of iterations, number of function evaluations, and CPU time. In the following, Figs 1, 2 and 3 represent the results presented in Tables 2, 3 and 4 in terms of NI, NF, and Time, respectively.

From Figs 1 and 2, we can conclude that Algorithm 4.1 performs better than Algorithm 4.2 and MPRP do in terms of the numbers of iterations and function evaluations. Moreover, the excellence of Algorithm 4.1 can obviously be attributed to the superior theoretical properties of the MHZ method compared with the usual HZ method. However, Fig 3 indicates that Algorithm 4.2 is superior to Algorithm 4.1 and MPRP in terms of CPU time. Overall, all methods are very effective for application to large-scale nonsmooth optimization problems.

## Conclusion

(i) In this paper, we focus on the HZ CG method and study the application of this method to solve nonsmooth optimization problems. Several results are presented that prove the efficiency of this method for application to large-scale problems of nonsmooth unconstrained optimization.

(ii) Motivated by the HZ formula, we also present a modified HZ CG formula. The modified HZ formula not only possesses the sufficient descent property of the HZ formula but also belongs to a trust region and has the non-negative scale $\beta_{k}^{GN}\geq 0.$

(iii) We report the results of applying three methods to solve large-scale nonsmooth convex minimization problems. Global convergence is achieved, and numerical experiments verify that both methods can be successfully used to solve large-scale nonsmooth problems.

(iv) Although the HZ and MHZ methods offer several key achievements for large-scale nonsmooth optimization, we believe that there are at least five issues that could be addressed to gain further improvements. The first is the scale *c* in the modified HZ CG algorithm, which could be adjusted. The second is the application of other CG methods for this type of optimization areas; perhaps there exists a more suitable CG method for this purpose. Regarding the third issue, it is well known that limited-memory quasi-Newton methods are effective techniques for solving certain classes of large-scale optimization problems because they require minimal storage; this inspires us to combine limited-memory quasi-Newton methods with the HZ CG technique to solve large-scale nonsmooth optimization. In future, we will also use the HZ CG method to investigate large-scale nonsmooth optimization with constraints; this is the fourth issue that we believe must be addressed. The last issue is the most important one, namely, the consideration of other optimality conditions and convergence conditions in nonsmooth problems should be paid. All of these issues will be addressed in our future work.
